# Heavy Metal Adsorption Using Magnetic Nanoparticles for Water Purification: A Critical Review

**DOI:** 10.3390/ma14247500

**Published:** 2021-12-07

**Authors:** Christos Liosis, Athina Papadopoulou, Evangelos Karvelas, Theodoros E. Karakasidis, Ioannis E. Sarris

**Affiliations:** 1 Department of Civil Engineering, University of Thessaly, 38334 Volos, Greece; cliosis@uth.gr; 2 Inorganic Chemistry Laboratory, Department of Chemistry, National and Kapodistrian University of Athens, 15771 Athens, Greece; athinapapad@chem.uoa.gr; 3 Department of Mechanical Engineering, University of West Attica, 12243 Athens, Greece; karvelas@uth.gr (E.K.); sarris@uniwa.gr (I.E.S.); 4 Condensed Matter Physics Lab, Department of Physics, University of Thessaly, 35100 Lamia, Greece

**Keywords:** adsorption, contamination, magnetic nanoparticles, heavy metals

## Abstract

Research on contamination of groundwater and drinking water is of major importance. Due to the rapid and significant progress in the last decade in nanotechnology and its potential applications to water purification, such as adsorption of heavy metal ion from contaminated water, a wide number of articles have been published. An evaluating frame of the main findings of recent research on heavy metal removal using magnetic nanoparticles, with emphasis on water quality and method applicability, is presented. A large number of articles have been studied with a focus on the synthesis and characterization procedures for bare and modified magnetic nanoparticles as well as on their adsorption capacity and the corresponding desorption process of the methods are presented. The present review analysis shows that the experimental procedures demonstrate high adsorption capacity for pollutants from aquatic solutions. Moreover, reuse of the employed nanoparticles up to five times leads to an efficiency up to 90%. We must mention also that in some rare occasions, nanoparticles have been reused up to 22 times.

## 1. Introduction

Nowadays, water issues, such as exhaustion of resources and quality of drinking water, have attracted the interest not only of researchers but also of national and international organizations and governments [1]. Terms such as water stress and water scarcity are now subjects of research on a daily basis. The first term refers to situations where the quantity of available water is not sufficient for agricultural, industrial, or domestic uses. It takes into account several physical aspects related to water resources, such as water quality, environmental flows, and water accessibility [2,3,4]. On the other hand, the second term refers to the volumetric abundance of water supply [5,6,7], which is the ratio of human water consumption to available water supply for a specific area [8,9]. The main factors that cause water crisis in the long run are population growth, expansion of industrial activities, urbanization, climate change, depletion of aquifers, and water contamination [10,11,12]. It is obvious that the coverage of global demands for safe drinking water in the near future is utopian, if we consider that water quantity is nearly constant due to the hydrologic cycle in various forms, such as seawater, groundwater, surface water, and rainwater [13,14]. However, climate change will affect the quality and quantity of potentially available drinking water due to increased flooding, more severe droughts, and enhanced toxicity of chemical contaminants in the environment [15,16]. Of more immediate concern is the efficiency of existing water treatment methods due to increasing pollution resulting from the anthropogenic activities [17]. Thus, purification of water from polluted sources is essential to enable the utilization of sustainable global water [18,19].

Water pollutants are categorized into point source, where the pollution originates from a single and identifiable source, and nonpoint source, where pollutants originate from a variety of sources [20]. Based on their main physicochemical characteristics, they can also be classified into radioactive, thermal, microorganism, nutrient, suspended solid and sediment, and organic and inorganic pollutants [21]. Inorganic pollutants consist of heavy metals, fertilizers, sulphides, ammonia, oxides of nitrogen, acids, and bases [22,23,24]. Since water quality is improving with increasing advances in technologies [17] and water purification is highly required to prevent toxic effects and disruption of ecological balance, this particular research review focuses on water contamination by heavy metals and ways of heavy metal removal.

Pollutants can contaminate water through both natural processes and anthropogenic activities [25,26]. Their concentrations in water depend on the local geological, hydrogeological, and geochemical characteristics of the aquifer. A significant burden on water quality and, consequently, public health is the increasing concentrations of heavy metals due to their toxicity, persistence, and bioaccumulative nature [25,27]. Unlike organic contaminants, heavy metals, which are toxic and mostly carcinogenetic, are not biodegradable and tend to accumulate in organisms [28]. Hence, exposure to toxic heavy metal through drinking water has long been a critical public health concern across the world [29]. Elements whose density exceeds 5 g/cm^3^ fall into the category of heavy metals [30] and are listed in Table 1 based on their toxicity, which is related to the maximum contaminant level (MCL) [31]. Specific differences in metal ion toxicities arise from differences in solubility, absorbability, transport, chemical reactivity, and the complexes that are formed within the body [32].

Heavy metals are classified into essential (Zn, Cu, Fe, and Co) and nonessential (Cd, Hg, As, and Cr) based on their toxicity. At low concentrations, essential heavy metals are harmless, unlike nonessential metals, which are highly toxic [34,35]. It is worth noting that for nonessential heavy metals, toxicity is very high, even at low concentrations [36,37]. Water contamination by heavy metals occurs in anthropogenic activities or in natural processes. Sources of contamination include metal corrosion, atmospheric deposition, soil erosion of metal ions, leaching of heavy metals, sediment resuspension, and metal evaporation from water resources to soil and groundwater. Phenomena such as weathering and volcanic eruptions fall into the category natural contamination [38]. In contrast, the major sources resulting from anthropogenic activities are mining wastes, landfill leaches, municipal wastewater, urban runoff, industrial wastewater, electroplating, and electronic and metal finishing industries [39,40]. Moreover, due to an increasing production of metals from technological activities, the problem of waste disposal has become of paramount importance; hence many aquatic environments face metal concentrations that do not meet water quality criteria designed to protect the environment, animals, and humans [41]. Metals such as Cu, Cd, Cr, Hg, and Ni have the ability to produce reactive radicals, resulting in DNA damage, lipid peroxidation, depletion of protein sulfhydryls, and other effects [42]. In this context, the availability and release of pollutants from anthropogenic sources needs to be considered [43]. The source of pollution and the effect of each heavy metal on human health are presented in Table 2.

However, the removal of heavy metal ions from water has been a vital and challenging issue for several decades [47,48], without reaching the heart of the problem. On the other hand, in recent years nanotechnology has been integrated with several novel techniques for the removal of heavy metals from water systems to improve removal efficiency [49,50,51].

Several water purification methods for heavy metals exist, such as chemical precipitation and coagulation, flocculation, electrochemical methods, photocatalytic degradation, membrane filtration, ion exchange, bioremediation, and adsorption, just to mention few [16,52,53]. The present work focuses on the adsorption methods developed during the last decade, where nanotechnology contributed quite a lot to great progress. In this context, adsorption capacity and reuse efficiency of magnetic nanoparticles for capturing heavy metals in water environments are investigated. In addition, the findings are analysed based on certain characteristics, such as viability method, time of purification, water quality after purification (adsorption capacity), and nanoparticle reusability.

The main interest in the adsorption method is the interaction between nanoparticles and adsorbents, which depends on their physicochemical properties [54]. The term physicochemical covers the particle size, surface area, surface charge, agglomeration, morphology, surface coating, and so forth. Particles with sizes below 100 nm are defined as nanoparticles [55], and their applications vary according to their size [54,56,57]. Magnetic, mechanical, optical, and electrical properties affect the formation and aggregation of nanoparticles [44]. Aggregation of nanoparticles is affected by surface charge, particle size, and composition. Nanoparticles without magnetic properties have limited applicability in water purification due to the difficulty of separation from the aqueous solution [58]. Magnetic nanoparticles present advantages due to their large surface area, size, and shape-dependent catalytic properties and can be separated from the aqueous solution with the use of a magnet field [48,59]; thus many methods have been investigated for their potential application in both environmental and biological fields [60,61,62].

Our focus was on studies that have been published during the last decade. The selection of relevant research articles was performed in several stages. In the first stage, the search was based on article title, abstract, and keywords. The terms for the search were (a) water purification, (b) magnetic nanoparticles, (c) heavy metals, and (d) reusability. In the second stage, we selected articles according to purification method (adsorption process). The final stage included the expansion of the bibliography using the remaining articles’ reference lists.

The sections below exhaustively describe the procedure for the adsorption method, which includes synthesis and characterization of the nanoparticles, the evaluation strategies, and the main findings, such as adsorption capacity and reusability.

## 2. Adsorption Method

The adsorption process occurs between a porous solid medium and a multicomponent fluid or gas by the formation of physical or chemical bonds and offers flexibility in design and operation, which is combined with very satisfying results (adsorption efficiency) [63]. Moreover, due to the reversibility of the adsorption mechanism, adsorbents can be regenerated through suitable desorption processes [28].

**Figure 1:**
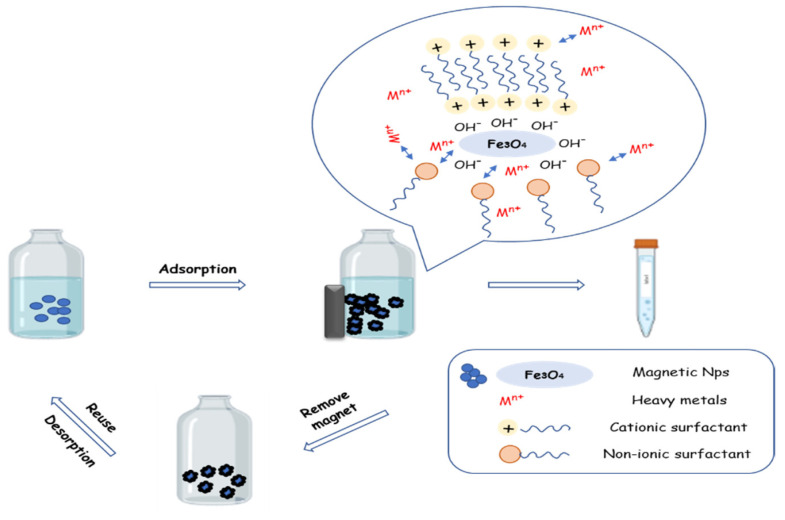
Schematic representation of the water purification cycle.

The term adsorption refers to a mass transfer process where the pollutants in a solution are transferred to a solid adsorbent [64], which is frequently used for water and wastewater purification under the principles of pore filling, H bonding, hydrophobic interaction, and ion exchange [65,66]. The process under physical or chemical techniques contains many different adsorption forces that can effectively adsorb specific pollutants [67]. Physical adsorption is due to weak Van der Waals forces of attraction, and chemical adsorption is due to the strong covalent bond between the adsorbent and the adsorbate [34]. In aqueous solutions, iron oxide hydroxyl is functionalized because of the Fe atoms that coordinate with water. Iron oxide surface atoms act as Lewis acids and coordinate with molecules that donate lone-pair electrons. These hydroxyl groups are amphoteric and may react with acids or bases [68,69]. Based on the physicochemical properties of magnetic nanoparticles and due to chemisorption and/or physisorption, we can achieve the adsorption of heavy metals from contaminated water. Terms such as adsorption capacity and removal efficiency are employed for the quantification of the adsorption process. The adsorption capacity is enhanced due to the increased surface atoms, which appear more active and unstable and offer more unsaturated bonds [67]. Unfortunately, the efficiency of conventional adsorbents is often limited by the surface area or active sites and the adsorption kinetics [66]. A schematic representation of the water purification cycle is presented in Figure 1.

**Figure 2:**
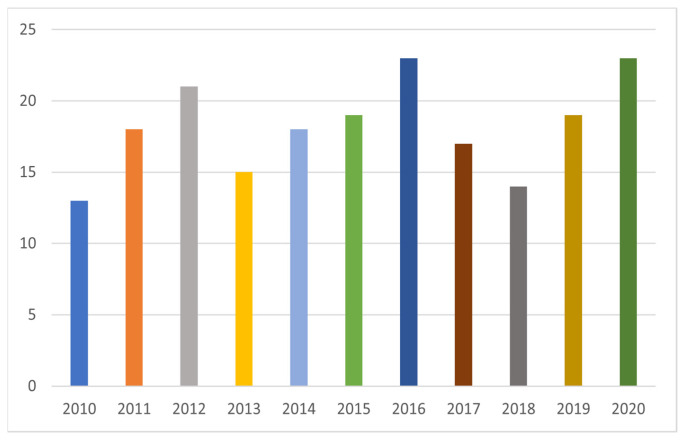
Selection of research articles per year.

**Figure 3:**
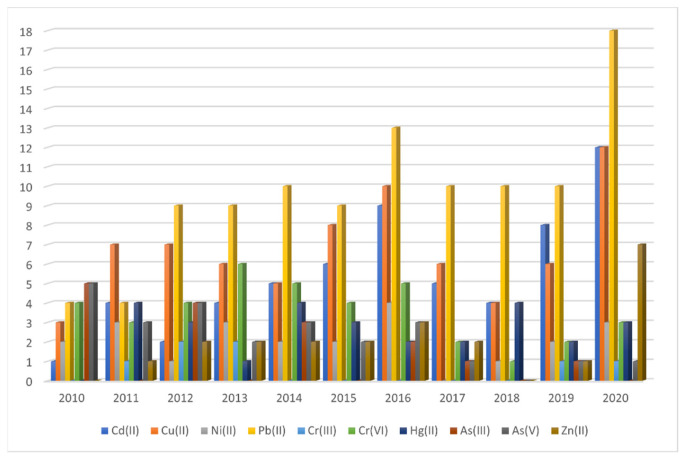
Research interest in each heavy metal per year since 2010.

Based on the selected articles, Figure 2 and Figure 3 were implemented. Despite the fact that the adsorption method of using bare or modified iron oxide nanoparticles in polluted aqua systems has already been used for several years, the scientific interest remains constant, as we can see in Figure 2. In Figure 3, we can see the research interest per year in each type of heavy metal ion. Furthermore, from Figure 3 we can see the total interest in each heavy metal during the last decade. It is evident that As (III) and Cr (III) constitute the minority of the heavy metals that have been studied by using the adsorption method. On the other hand, Pb (II), Cu (II), and Cr (VI) constitute the majority of the research interest, with Pb (II) appearing to have attracted the majority of the interest of the scientific community since it is discussed in almost 1/3 of the selected articles.

### 2.1. Synthesis and Characterization of Magnetic Nanoparticles

The adsorption method stages are synthesis, characterization of magnetic nanoparticles, and finally, evaluation of adsorption by specific methods, which are described below. Synthesis and characterization are correlated since the results of the initial stage, which is synthesis, are visualized and analysed through characterization.

Over the years, nanoparticle synthesis has been performed by top–down or bottom–up approaches with the use of chemical techniques, such as coprecipitation [70], microemulsions [71], sol–gel synthesis [72,73], sonochemical reactions [74], hydrothermal reactions [75], hydrolysis [76,77], thermolysis of precursors [78], flow injection [79,80], electrospray syntheses [81], solvothermal method [82], and laser ablation [83]

Moreover, several factors are affecting the size and the stability of nanoparticles, such as pH and temperature. Different sizes and shapes of nanoparticles could be synthesized by using the above synthesis methods. The average nanoparticle size of iron oxides increases with the increase in reaction temperature [84], and their size is estimated from either the Scherrer or Debye equation [85,86]. It is important to note that the Scherrer equation can only be applied for sizes between 100 and 200 nm [87,88].

The stabilization of iron oxide particles is crucial to obtain magnetic colloidal ferrofluids that are stable against aggregation in both a biological medium and a magnetic field [69]. In order to achieve stabilization, several coating methods [89,90] are used, such as monomeric stabilizers (carboxylates, phosphates) [91,92], inorganic materials (silica, gold, dextran), and polymeric stabilizers (chitosan, polyvinyl alcohol, polyethylene glycol, alginate) [93,94]. The functionalization of nanoparticles’ surface is of major importance for tuning the overall properties of particles to fit targeted applications [95].

A crucial factor is the surface charge of metal oxides due to the presence of hydroxyl groups depending on the pH of the solution (i.e., the surface of the magnetite will be positive or negative), the point of zero charge (pHpzc) caused by the amphoteric behaviour of hydroxylated surface groups, and the interaction between surface sites and the electrolyte species [96]. Therefore, the surface charge as well as the surface potential is essential in predicting surface reactions, surface complex formation, ion transfer, and so forth. Zeta potential, which reflects the potential difference between moving particles and the bulk phase, is a basic and major parameter to indicate the surface surroundings of a particle [97]. Hence, at the isoelectric point (PZC) particles flocculate because of the density of the surface charge, which is very small [98].

In addition, the efficiency of water purification techniques is strongly dependent on the efficiency of the adsorption and/or coprecipitation processes, which in turn are strongly influenced by the zeta potential of the colloidal substrate [99,100].

The ability and efficiency of the adsorption technologies in water treatment also depend on the characteristics and functions of adsorbents [101]. Another issue for magnetic nanoparticles at high temperatures is that the magnetic moments will rapidly fluctuate [102]. Generally, the saturation magnetization (MS) values found in nanostructured materials are usually smaller than the corresponding bulk phase, provided that no change in ionic configurations occurs [103]. The optimum saturated magnetization value could lead to a fast separation process, and the adsorbents could be easily separated from aqueous solution [104,105].

To obtain pure synthesized materials, they are isolated with the use of a magnetic field and, in almost all cases, washed with ultrapure water several times [106] and finally dried in a vacuum oven in order to remove redundant diamines [107].

The majority of existing research background focuses on the magnetic nanoparticles of iron oxide due to their superparamagnetic behaviour, high corrosion resistance [108], and low toxicity [109]. Moreover, composites of carbon and Fe_3_O_4_ exhibit excellent microwave absorption but also possess good conductivity and strength [110].

Hematite, magnetite, and maghemite excel significantly among iron oxides due to their unique magnetic, catalytic, and other properties [111,112]. Zerovalent metals are effectively reductant when reacting with oxidized pollutants in water [113]. Pollutant removal by γ-Fe_2_O_3_ nanoparticles has been recognized to be resulting from electrostatic interactions. Oxygen atoms on the surface of the iron oxides Fe_3_O_4_ and γ-Fe_2_O_3_ can be polarized under various pH values. More specifically, when the pH value is below the point of zero charge, the surface of the iron oxides possesses positive charges, attracting negatively charged pollutants [114,115]. The physical and magnetic properties of iron oxides are presented in Table 3.

Hematite is the most stable form of iron oxide polymorphs [117], and nanostructures can be obtained in various morphologies [118]; hence hematite is well fitted to the needs of each application. Hematite’s magnetic properties are related to its particle size: above the Neel temperature (T_N_) it is ferromagnetic, between T_N_ and T_M_ it is characterized by a reorientational magnetic phase transition, and below the Morin temperature (T_M_) it becomes antiferromagnetic [119].

Ferromagnetic iron oxides, such as magnetite with M_s_ 92–100 (emu/g) and magnemite with M_s_ 60–80 (emu/g), have a critical diameter value (D_cr_); below D_cr_, more energy is required to create a domain wall than to support the external magnetostatic energy of a single domain state [120]. The magnemite and magnetite nanoparticles become supermagnetic when the sizes of the nanoparticles are under 20 [121] and 25 nm, respectively. The above similar physical properties arise from the alike crystal structure. In general, as the size of the Fe_3_O_4_ nanoparticles decreases, the saturation magnetization also decreases [122]. In addition, magnetite iron oxide can be easily oxidized to Fe_2_O_3_ or dissolved in an acidic medium; therefore, anaerobic conditions during synthesis should be obtained in order to avoid oxidation [70]. In this context, it is obvious that plenty of factors are affecting the synthesis of magnetic nanoparticles, consequently raising the difficulty of the method. The sizes of the bare particles of hematite, magnetite, and maghemite under various synthesis methods and environments are presented in Table 4.

However, the use of magnetic nanoparticles, which provide larger removal capacity, higher reactivity, high surface-area-to-volume ratio, high degree of dispersion, excellent adsorption affinity, and catalytic activity [129], also presents a challenge in terms of recovery, stability, and toxicity. In chemical terms, iron oxide nanoparticles can be oxidized in air, resulting in the loss of dispersibility and magnetism [70].

Additionally, economic factors and the difficulty of separating them from the water phase make the use of pure iron oxide nanoparticles as an adsorbent dysfunctional [130]. Hence, surface modification could be the solution to these problems, offering protection of the magnetic core from dissolution in acid but also control over the agglomeration of magnetic nanoparticles. On the other hand, modification may have a negative impact on the activity of the particles, so modification approaches that can provide synergy between the physiochemical properties and the effectiveness of contaminant removal need further research [61,131,132]. In addition, low-cost absorbents with high metal-binding capacity are increasingly being utilized for the removal of heavy metals.

The characterization of nanoparticles is a major part of the method [133], for having the optimum adsorption capacity, the nanoparticles must be well defined in frames that depend on several parameters. These parameters determine and affect the nanoparticles’ applicability, such as size, shape, size distribution, degree of aggregation, surface charge, and surface area [134,135,136,137]. The most common characterization method for nanoparticles of <100 nm size is transmission electron microscopy (TEM), which is used for the characterization mostly of the size, shape, and dispersion of magnetic nanoparticles but also for shape heterogeneity and accumulation [133,134,138]. The size distribution of the nanoparticles can be measured by a dynamic laser scattering analyser (DLS) [136,138,139,140]. The identification of the functional groups in the synthesized magnetic nanoparticles can be obtained by Fourier-transform infrared spectroscopy (FTIR) technique. The absorption spectra of the nanoparticles were determined using ultraviolet–visible (UV–VIS) spectroscopy [141,142]. Zeta potential measurement is used for the determination of the surface charge of nanoparticles. In general, nanoparticles with zeta potential values greater than the absolute value of 30 mv present good stability [143]. X-ray diffraction (XRD) is used for structural characterization, such as structural determination, shape, and size [110,144]. Magnetic force microscopy (MFM) is used for the investigation of the magnetic behaviour with high spatial resolution for static magnetic fields [143,145,146].

### 2.2. Adsorption Evaluation Strategies

Characterization methods are necessary for the evaluation of the adsorption. Among the most widely employed methods are Fourier-transform infrared spectroscopy (FTIR) technique, ultraviolet–visible (UV–VIS) spectroscopy, ICP analysis, and magnetic property measurement by a VSM (vibrating sample magnetometer).

Apart from the adsorption capacity, further analysis of the adsorption process performance is obtained from the thermodynamic and kinetic aspects [147]. The adsorption method could be separated in reaction and diffusion models; for the examination of these two models, several methods have been developed.

The pollutant removal efficiency R and adsorption capacity q are calculated using Equations (1) and (2):

(1) $$R=\left(C_{0}-\mathrm{Ce}\right)\times 100/C_{0}$$

(2) $$q=\left(C_{0}-C\right)\times V/M$$

where C_0_ (mg/L) and C (mg/L) are the initial and final pollutant concentrations in the solution, respectively. V(L) is the volume of the solution, and M(g) is the mass of the magnetic nanoparticles [148].

When an adsorbate-containing phase is contacted with the adsorbent for sufficient time, then the adsorption equilibrium is established [149].

The equilibrium correlations and performance are described by the interactions between adsorbents and pollutants, which are known as adsorption isotherms [63]. These isotherms provide plenty of information that, if utilized properly, permits the optimization of the adsorption mechanism pathways, the expression of the surface properties and capacities of adsorbents, and the effective design of the adsorption systems [150]. Sometimes it is difficult to estimate whether the equilibrium has been reached due to the kinetic hysteresis that occurs when a fast kinetic adsorption reaction is followed by slower adsorptions. The two parameters of the Langmuir adsorption isotherm are used for the analysis of the adsorption process according to Equation (3) [151,152,153]:

(3) $$q_{e}=q_{m}\times K_{L}C_{e}/\left(1+K_{L}C_{e}\right)$$

where q_e_ (mg/g) is the equilibrium adsorption capacity of the heavy metal ions, q_m_ (mg/g) is the maximum adsorption capacity of the heavy metal ions, C_e_ (mg/L) is the equilibrium concentration of the ions, and K_L_ is the Langmuir adsorption constant. The two-parameter isotherm Freundlich model is employed also according to Equation (4) [65,151,152]:

(4) $$q_{e}=K_{F}\times \left(C_{e}^{\frac{1}{n}}\right)$$

where q_e_ (mg/g) is the equilibrium adsorption capacity of the heavy metal ions, q_m_ (mg/g) is the maximum adsorption capacity of the heavy metal ions, C_e_ (mg/L) is the equilibrium concentration of the ions, K_F_ is the Freundlich constant indicating the adsorption capacity, and n is the heterogeneity factor representing the adsorption intensity.

The limitations of the Freundlich isotherm model arise from the fact that it is an empirical model, its behaviour is linear only for certain concentrations, and finally, the constant K_F_ varies with the change of temperature [154].

Another isotherm model arises from the combination of Langmuir and Freundlich expressions for the prediction of the heterogeneous adsorption systems [155], the so-called Sips model, which is described by Equation (5) [65,151,156]:

(5) $$q_{e}=q_{s}\times K_{s}C_{e}^{m}/\left(1+K_{s}C_{e}^{m}\right)$$

where q_s_ (mL/mg) is the specific adsorption capacity at saturation, K_s_ (mL/mg) is the Sips isotherm constant, and m is the heterogeneity factor. The majority of the studied research works have employed the isotherm models described above.

The adsorption mechanism and the metal ion uptake rate, to establish the time dependence of the residual adsorbate in solution, are provided by the kinetic analysis of the adsorption process [157]. Several models have been employed to describe the kinetics, but the pseudo first order and the pseudo second order have dominated [158] in the existing research field for the adsorption reaction model. The linearized pseudo first order was introduced by Lagergren (1898) and is described by Equation (6) [159,160]:

(6) $$\ln\left(q_{e}-q\left(t\right)\right)=\ln q_{e}-K_{1}\times t$$

where q_t_ and q_e_ are the amounts of adsorption at time *t* and equilibrium per unit mass of adsorbent mg/g, respectively, and k_1_ is the pseudo first-order rate constant 1/h. The transformed linear pseudo second order is given by Equation (7) [159,160,161]:

(7) $$\frac{t}{q\left(t\right)}=\frac{1}{K_{2}q_{e}^{2}}-\frac{t}{q_{e}}$$

where q(t) and q_e_ are the amount of adsorption at time t and equilibrium per unit mass of adsorbent mg/g, respectively, and k_2_ is the pseudo second-order rate constant g/mg/h. The pseudo first order and the pseudo second order are empirical models, and because of that, the physical meanings cannot investigate the mass transfer mechanisms [162].

Thermodynamic analysis determines the feasibility, spontaneity, and heat change of the adsorption process [160]. The thermodynamic equations given by Van’t Hoff are described by Equations (8) and (9) [159,160,163]:

(8) $${\Delta G}^{0}=-\mathrm{RT}\ln K_{L}$$

(9) $${\Delta G}^{0}={\Delta H}^{0}-T{\Delta S}^{0}$$

where the parameters are the free energy (Gibbs) change ΔG^0^, the enthalpy change ΔH^0^, and the entropy change ΔS^0^ and are estimated from the change in the thermodynamic equilibrium. Additionally, R (8.314 J/mol × K) is the gas constant, T (°K) is the absolute temperature, and K_L_ (L/mol) is the Langmuir constant. The exothermic reaction of the adsorption is confirmed by the negative values of the enthalpy, which also indicate decreased randomness at the solid/solution interface with some structural changes in the adsorbate and adsorbent [164].

From the above mathematical analysis of the adsorption process, it can be understood that in order to extract safe results from the experiments, the evaluation strategies should be meticulously followed. In summary, the adsorption isotherms are employed for the evaluation of the adsorption capacity and the investigation of the characteristics of adsorption, while the pseudo first- and second-order equations are used to illustrate the adsorption kinetics of the adsorbent surfaces [165].

## 3. Main Findings during the Last Decade

Restrictions on the use of bare hematite, magnetite, and maghemite nanoparticles force the majority of researchers to synthesize modified nanoparticles from iron oxide. A large number of parameters apart from those of the magnetic nanoparticle synthesis, which have been analysed above, affect the adsorption efficiency of heavy metals, among them being pH, contact time, temperature, adsorbent dose, and initial ion concentration [58,166]. pH is a factor that is involved not only at this stage of the method but also during the synthesis of nanoparticles; almost in all cases, the pH values are different between these two stages. pH is directly related to the competition ability of hydrogen and metal ions to the adsorbent surface active sites, for by increasing the pH value due to the formation of soluble hydroxylated metal complexes, the metal uptake capacity decreases [161], the maximum adsorption capacity that is observed varies from pH 2 to 9, and most researchers achieve optimum adsorption capacity at pH 5–7. The adsorbent dosage is directly related to the adsorbent capacity since it determines the contact areas between the adsorbent and the adsorbate [167]. Moreover, when the adsorbent dose is increasing, the number of available binding sites is also increasing; but as the equilibrium point of adsorption is reached, the efficiency does not reflect the available sites and remains steady [161]. A critical point also exists for the contact time parameter, since initially, removal rates increase rapidly with time; then they gradually decrease due to the availability of the binding sites until the equilibrium is reached [161]. Experiments show that even for the same adsorbent but for different pollutants, the ideal contact time may vary significantly, as in some cases it is between 30 min to 12 h [157]. The factor that has been investigated less is the effect of coexisting cations/anions, as in previous parameters, the initial ion concentration does not differ; thus removal rates increase with increasing initial concentrations until a point where the rates remain unaffected [161]. Research shows that these cations/anions that are contained in water have no significant influence on adsorption capacity [168,169], since the adsorbent surfaces suggest multisurface adsorption active sites. Moreover, differences in the radius of heavy metal ions have significant influence on adsorption efficiency [170], ions with a smaller radius have higher mobility in aqueous solutions, and therefore, they have a lower tendency to adsorb on magnetic nanoparticles. Additionally, the temperature that is used during the adsorption process varies between 15 and 45 °C, although in some rare occasions, it reaches 70 °C.

Every research method must be evaluated according to the criteria that have been set from the beginning. At this phase of the literature review, the results of heavy metal adsorption in aquatic solutions using bare or modified magnetic iron oxide nanoparticles are summarized. In order to be able to evaluate the methods through the experimental results since 2010, many factors must be taken into consideration. Initially, many researchers used different nanoparticle compounds for various pollutants, but also observed a large selectivity of the initial parameters (i.e., adsorbent dosage, contact time, pH, temperature), which creates difficulties in the categorization of the experimental results. Despite that, the main purpose of every work is the removal of heavy metals; thus adsorption capacity is the main factor that has been focused on in each research. Hence, Table 5 provides useful information, such as the time of equilibrium/contact time, pH, adsorption capacity or removal efficiency, and temperature during the adsorption process, which could lead to safe extractions for the applicability of the method, which is based on the findings during the last decade. Additionally, the findings are listed in chronological order. Due to several nanoparticle compounds, the equilibrium time is not constant but has large dispersion.

A crucial stage in the evaluation of the applicability of the experimental methods is the possibility of reducing the production cost and time. This reduction could be achieved by reusing the existing nanoparticles; thus the stages of synthesis and characterization could be avoided. Desorption processes may occur either by thermal treatment or through suitable desorbing agents and are necessary for recycling [171], so the nanoparticles could be used again. Among the selected articles, all have employed desorbing agents during the desorption process. The performance of the desorption process for magnetic nanoparticles is directly related to the size, coating, magnetic behaviour [159] and pH of the solution [357], while other critical factors are the kind of regenerative solutions (i.e., NaOH, HCl) but also their concentration. For example, 2 M of HNO_3_ has a desorption efficiency of Cr (VI) equal to 73%, but the efficiency drops to 20% when 0.1 M of HNO_3_ is employed [231]. Additional benefits of the desorption process are the limited cost of desorbing agents and the time of the process, which could be achieved in less than 1 h. The usage of the desorption process is enhanced by the ease of collection, which comes from the selectivity of the paramagnetic nanoparticles assisting the technique, because they could be readily separated from the solution when a magnetic field is applied; thus iron oxide nanoparticles are more preferable to other nanoparticles with no magnetic cores. Additionally, computational fluid dynamics could be employed at this stage, reducing the cost of the materials. The major advantage of computational water treatment methods compared with an experimental method is that the steps of synthesis and characterization of magnetic nanoparticles are not time-consuming since they do not exist. The aims of microfluidic mixing and driving simulations for water purification from heavy metal ions are to achieve rapid mixing and desired guidance of nanoparticles [48,51,59,358].

Nonetheless, the effectiveness of the adsorption–desorption process is evaluated by the efficiency of heavy metal adsorption after each recycling. An ideal adsorbent is considered to be one that simultaneously possesses high adsorption capability and high desorption efficiency [359]. Of critical importance is the effectiveness of the process in several studies, in which up to five cycles of adsorption without a significant decrease in efficiency have been achieved [360]. In addition, high adsorption and desorption efficiencies equal to 98.4% after seven cycles [306] and 98% (constant) for over 15 cycles have been reported [171]. The deterioration of active binding sites on the surface absorbent during recycling results in a decrease in efficiency. It should be noted, however, that in some cases after the desorption process, the adsorption efficiency did not decrease, but instead, it increased in the next cycle [361]; this phenomenon is based on the increase in the positively charged surface of nanoparticles, which leads to increased electrostatic attraction forces between the iron oxide nanoparticles and the pollutant. Recycling of the adsorbent is important to obtain the process that enhances the viability of the adsorption method.

The recycling efficiency and adsorption capacity for each cycle are presented in Table 6 and Table 7, respectively. We must mention here that there are very few articles that have investigated the adsorption capacity. This fact has a negative impact on the applicability of the method. During the literature review were recorded cases where the recycling effectiveness was measured with adsorption capacity instead of efficiency. Additionally, both adsorption and desorption efficiency decreased through the regeneration cycles and due to the difficulty in reversing adsorption [362].

## 4. Conclusions

During the last decade, nanotechnology has led to great progress in several fields, including water purification and heavy metal removal, with a large number of published articles. Therefore, a need arises for a frame of water purification from heavy metals using the adsorption method, which includes not only the main findings during the last decade but also all the phases of the technique. The success of the method is based not only on adsorption efficiency or capacity but also on applicability.

We must consider that a specific bare or modified nanoparticle that can act as a panacea for water purification from all heavy metals does not exist. This is proved by the experimental results, which show that adsorption capacity differs between pollutants, while all the other parameters remain constant. However, according to the literature review, researchers seem to have investigated more capping agents, such as SiO_2_, amino groups, and graphene oxide. Additionally, the removal efficiency is different for various water sources (lake, river, groundwater, tap water, and sea) under the same heavy metal ion and adsorbent [187]. Moreover, from the context that is delimited above, it is clear that for each parameter exists a critical point where the adsorption efficiency is reaching a maximum, resulting in difficulty in scaling up. It is clear from the equilibrium time that the scale-up could be more realistic in reservoirs rather than in the case of continuous flow inside pipes. The ideal range of pH for the maximum adsorption without dependence on the pollutant is between 5 and 7. However, the main findings that are presented in this review almost reach the optimum efficiency. An encouraging fact is that the majority of experiments take place at room temperature with high adsorption capacity. The ability to reuse nanoparticles after desorption constitutes a significant parameter of applicability. Therefore, the preparation of each adsorbent must be targeted for the removal of a specific pollutant by the adsorption method.

## Figures and Tables

**Table 1:** Metal toxicity [31,33].

Heavy Metals	MCL (mg/L) × 10^2^
Zinc (Zn)	80
Nickel (Ni)	20
Copper (Cu)	25
Chromium (Cr)	5
Arsenic (As)	5
Cadmium (Cd)	1
Lead (Pb)	0.06
Mercury (Hg)	0.003

**Table 2:** Heavy metal characteristics [16,33,44,45,46].

Heavy Metal	Human Health Impacts	Common Sources
Arsenic	Skin damage, circulatory system issues, protein coagulation, nerve inflammation, muscle weakness, carcinogenicity	Naturally occurring, electronic production, agricultural applications, nonferrous smelters, metallurgy, coal-fired and geothermal electrical generation, tanning, pigments, antifouling paints, light filters, fireworks, veterinary medicine
Cadmium	Kidney damage, carcinogenicity, DNA damage, gastrointestinal irritation, hyperactivity, renal failure	Naturally occurring, various chemical industries, agricultural applications (phosphatic fertilizers), pigments, anticorrosive metal coatings, plastic stabilizers, alloys, coal combustion
Chromium	Allergic dermatitis, diarrhoea, nausea, vomiting, headache, neurotoxicity, kidney and liver damage	Naturally occurring, steel manufacturing metallurgy, refractory, chemical industries, plating, pigments, textile and leather tanning, passivation of corrosion of cooling circuits, wood treatment
Copper	Gastrointestinal issues, liver and kidney damage, anorexia, Wilson’s disease	Household plumbing systems, naturally occurring, chemical and pharmaceutical equipment, pigments, alloys
Lead	Kidney damage, reduced neural development, carcinogenicity, high blood pressure	Lead-based products (batteries), household plumbing systems, antiknock agents, pigments, glassware, ceramics, plastic, alloys, sheets, cable sheathings, solder
Mercury	Kidney damage, nervous system damage, carcinogenicity, gingivitis, stomatitis, gastrointestinal issues, abortions	Fossil fuel combustion, electronic industries, fluorescent light bulbs, electrical and measuring apparatus, catalysts, pharmaceuticals, dental fillings, scientific instruments, rectifiers, oscillators, solders
Nickel	Allergic dermatitis, nausea, chronic asthma, coughing, carcinogenicity, hair loss	Paper products, fertilizer plating, electroplating, batteries, arc welding, rods, pigments for paints and ceramics, surgical and dental prostheses, moulds for ceramic and glass containers, computer components, catalysts
Zinc	Depression, lethargy, neurological signs, increased thirst, hyperactivity, physical dysfunction	Mining, coal, waste combustion, steel processing, agricultural applications (phosphatic fertilizers), anticorrosion coating, batteries, cans, PVC stabilizers, medicines and chemicals, rubber industry, paints, soldering and welding fluxes

**Table 3:** Physical and magnetic properties of iron oxide [116,117].

Molecular Formula	α-Fe_2_O_3_	Fe_3_O_4_	γ-Fe_2_O_3_
Density (g/cm^3^)	5.26	5.18	4.87
Melting point (°C) Hardness	1350	1583–1597	-
	6.5	5.5	5
Type of magnetism	Weakly ferromagnetic, antiferromagnetic	Ferromagnetic	Ferromagnetic
Curie temperature (K)	-	850	948
Point of zero charge (pHpzc)	-	-	7.5
Morin temperature (K)	263	-	-
Neel temperature (K)	948 < T_N_ < 963	-	-
a	- Rhombohedral	0.8394 Cubic	0.8346 Cubic

**Table 4:** Synthesis methods for bare iron oxide nanoparticles’ sizes under various conditions.

Method	Iron Oxide	Nanoparticle Size (nm)	pH	Temperature (°C)	Reference
Coprecipitation	Fe_3_O_4_	8.3	11	45	[86]
Sonochemical coprecipitation (ultrasound assistance)	Fe_3_O_4_	13			[74]
Sonochemical coprecipitation	Fe_3_O_4_	17			[74]
Coprecipitation	Fe_3_O_4_	10–20	9	80	[123]
Ultrasonic-assisted chemical coprecipitation	Fe_3_O_4_	15	7	60	[124]
Coprecipitation	Fe_3_O_4_	13.2	11	85	[86]
Sol–gel	Fe_3_O_4_	2.02		200	[73]
Sol–gel	Fe_3_O_4_	5.58		400	[73]
Sol–gel	Fe_3_O_4_	8.35		600	[73]
	Fe_3_O_4_	23			[125]
	γ-Fe_2_O_3_	2		130	[84]
	γ-Fe_2_O_3_	4.5		180	[84]
	γ-Fe_2_O_3_	6.1		200	[84]
	γ-Fe_2_O_3_	9		230	[84]
	γ-Fe_2_O_3_	12		250	[84]
	γ-Fe_2_O_3_	25.5		250	[125]
Solvothermal	γ-Fe_2_O_3_	3000		400	[126]
Mechanochemical milling	Fe_2_O_3_	10,000			[127]
Mechanochemical milling	α-Fe_2_O_3_	17.1			[128]
Ultrasonic spray pyrolysis	α-Fe_2_O_3_	18		400	[118]
Ultrasonic spray pyrolysis	α-Fe_2_O_3_	33		600	[118]
	α-Fe_2_O_3_	53.7		500	[125]

**Table 5:** Adsorption capacity, removal efficiency, and system conditions.

Year of Publish	Magnetic Nanoparticle	Heavy Metal Ion	Adsorption Capacity (mg/g) ^1^	Removal Efficiency (%)	Time (min)	pH	Tempe-rature (°C)	Number of Cycles	Ref.
2010	Amino-functionalized Fe_3_O_4_	Cu(II)	25.77		5	6	25	15	[171]
	Fe_3_O_4_	As(V)	44.1			3	25		[169]
	Fe_3_O_4_	As(III)	49.8			7	25		[169]
	MnFe_2_O_4_	As(V)	90.4			3	25		[169]
	MnFe_2_O_4_	As(III)	93.8			7	25		[169]
	CoFe_2_O_4_	As(V)	73.8			3	25		[169]
	CoFe_2_O_4_	As(III)	100.3			7	25		[169]
	Amino-functionalized Fe_3_O_4_@SiO_2_	Cu(II)	0.69 mmol/g				45	4	[172]
	Amino-functionalized Fe_3_O_4_@SiO_2_	Cu(II)	0.60 mmol/g				35	4	[172]
	Amino-functionalized Fe_3_O_4_@SiO_2_	Cu(II)	0.47 mmol/g				25	4	[172]
	Amino-functionalized Fe_3_O_4_@SiO_2_	Pb(II)	0.54 mmol/g				45	4	[172]
	Amino-functionalized Fe_3_O_4_@SiO_2_	Pb(II)	0.45 mmol/g				35	4	[172]
2010	Amino-functionalized Fe_3_O_4_@SiO_2_	Pb(II)	0.37 mmol/g				25	4	[172]
	Amino-functionalized Fe_3_O_4_@SiO_2_	Cd(II)	0.33 mmol/g				45	4	[172]
	Amino-functionalized Fe_3_O_4_@SiO_2_	Cd(II)	0.27 mmol/g				35	4	[172]
	Amino-functionalized Fe_3_O_4_@SiO_2_	Cd(II)	0.20 mmol/g				25	4	[172]
	GA–APTES-NPs	Cu(II)	61.07	75.3	15	4–5.3	20 ± 0.1	3	[173]
	Fe_3_O_4_	Pb(II)	36		30	5.5		5	[168]
	EDA-MPs-10	Cr(VI)	61.35		60	2.5	35		[107]
	EDA-MPs-8	Cr(VI)	60.98		60	2.5	35		[107]
	EDA-MPs-6	Cr(VI)	49.5		60	2.5	35		[107]
	EDA-MPs-4	Cr(VI)	36.63		60	2.5	35		[107]
	EDA-MPs-2	Cr(VI)	32.15		60	2.5	35		[107]
	Fe_3_O_4_-γ-Fe_2_O_3_	As(III)	3.69	96		2			[115]
	Fe_3_O_4_-γ-Fe_2_O_3_	As(V)	3.71	96		2			[115]
	Fe_3_O_4_-γ-Fe_2_O_3_	Cr(VI)	2.4	99		2			[115]
	Fe_3_O_4_	Pb(II)	63.33			6			[174]
	Fe_3_O_4_	Ni(II)	52.55			6			[174]
	Fe_3_O_4_@SiO_2_	Pb(II)		97.34		6		5	[175]
	Nanoiron	Ni(II)	11.53			5	25		[176]
	EDA-NMPs	Cr(VI)	136.98		30	2.5	35		[177]
	DEDA-NMPs	Cr(VI)	149.25		30	2.5	35		[177]
	TETA-NMPs	Cr(VI)	204.08		30	2.5	35		[177]
	TEPA-NMPs	Cr(VI)	370.37		30	2	35		[177]
	Magnetite NPs	Cr(VI)		82	20	2			[106]
	Fe_3_O_4_-γFe2O_3_	As(ΙΙΙ)	4.75	91	180	6.5			[178]
	Fe_3_O_4_-γFe_2_O_3_	As(V)	4.85	92	180	6.5			[178]
2011	γ-Fe_2_O_3_	Hg(ΙΙ)	140			8			[157]
	Iron oxide-coated perlite (IOCP)	As(V)	0.39		5	6.5–7			[165]
	γ-Fe_2_O_3_ onto ball-milled expanded perlite carrier	As(V)	4.64			7			[179]
	NiFe_2_O_4_	Cu(II)	55.83						[180]
	NiFe_2_O_4_	Cr(VI)	36.95						[180]
	NiFe_2_O_4_	Ni(II)	37.02						[180]
	EDTAD-treated Fe_3_O_4_	Pb(II)	99.26			5.5	30		[181]
	EDTAD-treated Fe_3_O_4_	Cd(II)	48.70			6	30		[181]
	Fe_3_O_4_@SiO_2_-MIIP	Cu(II)	24.2					5	[182]
	Fe_3_O_4_@SiO_2_-NIP	Cu(II)	5.2						[182]
	Fe_3_O_4_–TW	Ni(II)	38.3					5	[183]
	γ-Fe_2_O_3_@Fe_3_O_4_	Cr(VI)	74.07		30		35		[184]
	γ-Fe_2_O_3_@Fe_3_O_4_	Cr(VI)	78.13		30		25		[184]
	γ-Fe_2_O_3_@Fe_3_O_4_	Cr(VI)	83.33		30		15		[184]
	Polyrhodanine-coated γ-Fe_2_O_3_	Hg(II)	179					5	[157]
	Polypyrrole/F_3_O_4_	Cr(VI)	169.49		30–180	2	25	3	[185]
	Polypyrrole/F_3_O_4_	Cr(VI)	204.08		30–180	2	35		[185]
2011	Polypyrrole/F_3_O_4_	Cr(VI)	238.09		30–180	2	45		[185]
	Iron oxide-modified sewage sludge	Pb(II)	42.4			6	25 ± 0.1		[186]
	Iron oxide-modified sewage sludge	Cu(II)	17.3			6	25 ± 0.1		[186]
	Iron oxide-modified sewage sludge	Cd(II)	14.7			7	25 ± 0.1		[186]
	Iron oxide-modified sewage sludge	Ni(II)	7.8			7	25 ± 0.1		[186]
	SH-mSi@Fe_3_O_4_	Hg(II)	260			6.5	25	6	[187]
	SH-mSi@Fe_3_O_4_	Pb(II)	91.5			6.5	25	6	[187]
	Fe_3_O_4_@SiO_2_	Hg(II)		98					[188]
	MWCNT/IO/CD	Cu(II)		59		5.5	25.15		[189]
	CS-co-MMB-co-PAA hydrogel	Pb(II)	163.90			5.5	25		[190]
	CS-co-MMB-co-PAA hydrogel	Cd(II)	135.51			5.5	25		[190]
	CS-co-MMB-co-PAA hydrogel	Cu(II)	152.42			5.5	25		[190]
	MWCNT/nano-iron oxide	Cr(III)		82		5			[191]
	MWCNT/nano-iron oxide	Cr(III)		88		6			[191]
	Nano-Fe_3_O_4_	Cu(II)	8.90			5	25		[192]
	PEI-grafted magnetic porous	Cu(II)	157.8		10	6–7.5		4	[193]
	PEI-grafted magnetic porous	Zn(II)	138.8		10	6–7.5		4	[193]
	PEI-grafted magnetic porous	Cd(II)	105.2		10	6–7.5		4	[193]
	Fe_3_O_4_-coated boron nitride nanotubes	As(V)	0.96		720	9	25	4	[194]
2012	Fe_3_O_4_-C	Pb(II)	126			6			[195]
	Pectin-coated iron oxide	Cu(II)	48.9						[196]
	Fe_3_O_4_@ZrO_2_	Cr(III)	24.5			8–9			[131]
	MNPs–Ca-alginate immobilized P. chrysosporium	Pb(II)	176.33				35	5	[197]
	AF-Fe_3_O_4_	Cu(II)	523.6		120	7			[198]
	AF-Fe_3_O_4_	Cd(II)	446.4		120	7			[198]
	AF-Fe_3_O_4_	Pb(II)	369.0		120	7			[198]
	Fe_3_O_4_@APS@AA-co-CA	Cd(II)	29.6		45	5.5	25	4	[199]
	Fe_3_O_4_@APS@AA-co-CA	Zn(II)	43.4		45	5.5	25	4	[199]
	Fe_3_O_4_@APS@AA-co-CA	Pb(II)	166.1		45	5.5	25	4	[199]
	Fe_3_O_4_@APS@AA-co-CA	Cu(II)	126.9		45	5.5	25	4	[199]
	Fe_3_O_4_–SiO_2_-poly	As(III)	84±5		120	6	30		[104]
	Fe_3_O_4_–SiO_2_-poly	Cu(II)	65±3		120	6	30		[104]
	Fe_3_O_4_–SiO_2_-poly	Cr(III)	77±3		120	5.3	30		[104]
	Acid-coated Fe_3_O_4_	As(V)	16.56						[200]
	Acid-coated Fe_3_O_4_	As(III)	46.06						[200]
	γ-Fe_2_O_3_ functionalized with citrate ions	Ni(II)	0.57 mmol/g		15	6 ± 0.5			[201]
	Fe_3_O_4_@CTAB	As(V)	23.07		2	6		5	[202]
	Fe_3_O_4_-γ-Fe_2_O_3_	Cr(VI)	6			4	10		[202]
2012	Fe_3_O_4_-γ-Fe_2_O_3_	Cr(VI)	6.9			4	22		[202]
	Fe_3_O_4_-γ-Fe_2_O_3_	Cr(VI)	7			4	50		[202]
	Fe_3_O_4_–PEI800–MMT	Cr(VI)	8.77						[203]
	Fe_3_O_4_–PEI25000–MMT	Cr(VI)	7.69						[203]
	MPTS-CNTs/Fe_3_O_4_	Hg(II)	65.52			6.5	25 ± 0.2		[204]
	MPTS-CNTs/Fe_3_O_4_	Pb(II)	65.40			6.5	25 ± 0.2		[204]
	rGO–Fe(0)–Fe_3_O_4_	As(III)	44		60	7	25		[205]
	rGO–Fe_3_O_4_	As(III)	21		60	7	25		[205]
	Nanomagnetite (NMT)	Cu(II)	14.3		70		45		[206]
	Nanomagnetite (NMT)	As(V)	6.5		120		45		[206]
	Water-soluble Fe_3_O_4_ nanoparticles	Pb(II)	96.8		60	7	18		[207]
	Water-soluble Fe_3_O_4_ nanoparticles	Cr(VI)	41.5		90	7	18		[207]
	TF-SCMNPs	Hg(II)	207	93.76	15	6	22.5		[208]
	M-MIONPs	Hg(II)		98.6	4	9	25		[209]
	Fe_3_O_4_-RGO–MnO_2_	As(III)	14.04			7	25		[210]
	Fe_3_O_4_-RGO–MnO_2_	As(V)	12.22			7	25		[210]
	MWCNTs/Fe_3_O_4_	Pb(II)	41.77			5.3		5	[211]
	MWCNTs/Fe_3_O_4_-NH_2_	Pb(II)	75.02			5.3		5	[211]
	γ-Fe_2_O_3_	Cu(II)	71.42			6 ± 0.1	25 ± 1		[212]
	γ-Fe_2_O_3_	Zn(II)	111.11			6 ± 0.1	25 ± 1		[212]
	γ-Fe_2_O_3_	Pb(II)	84.95			6 ± 0.1	25 ± 1		[212]
	Fe_3_O_4_/CS/PAA	Cu(II)	193						[213]
2013	Fe_3_O_4_/SiO_2_	Pb(II)	14.65			4	27		[214]
	Fe_3_O_4_/SiO_2_	Pb(II)	16.83			4	50		[214]
	Fe_3_O_4_/SiO_2_	Pb(II)	17.65			4	70		[214]
	Fe_2_O_3_–Al2O_3_	Cu(II)	4.98		60	6		4	[215]
	Fe_2_O_3_–Al_2_O_3_	Pb(II)	23.75		60	6		4	[215]
	Fe_2_O_3_–Al_2_O_3_	Ni(II)	32.36		60	6		4	[215]
	Fe_2_O_3_–Al_2_O_3_	Hg(II)	63.69		60	6		4	[215]
	Mixed magnetite– hematite	Pb(II)	617.3		60	7	25		[216]
	Mixed magnetite– hematite	Cr(III)	277.0		120	7	25		[216]
	Mixed magnetite– hematite	Cd(II)	223.7		1440	7	25		[216]
	Chitosan-coated MnFe_2_O_4_ (CCMNPs)	Cu(II)	22.6			6		5	[217]
	Chitosan-coated MnFe_2_O_4_ (CCMNPs)	Cr(VI)	15.4			6		5	[217]
	γ-PGA/Fe_3_O_4_ MNPs	Cr(III)	162.6	99.66	120	6	30		[218]
	CDpoly-MNPs	Pb(II)	64.5		45	5.5–6	25	4	[219]
	CDpoly-MNPs	Cd(II)	27.7		45	5.5–6	25		[219]
	CDpoly-MNPs	Ni(II)	13.2		45	5.5–6	25		[219]
	3D flowerlike a-Fe_2_O_3_	As(V)	41.46		120				[220]
	3D flowerlike a-Fe_2_O_3_	Cr(VI)	33.82		120				[220]
	Magnetite nanorods	Pb(II)	112.86		60	5–6	25		[221]
	Magnetite nanorods	Zn(II)	107.27		60	5–6	25		[221]
2013	Magnetite nanorods	Ni(II)	95.42		60	5–6	25		[221]
	Magnetite nanorods	Cd(II)	88.39		60	5–6	25		[221]
	Magnetite nanorods	Cu(II)	76.10		60	5–6	25		[221]
	Maghemite nanotubes	Pb(II)	71.42						[221]
	Maghemite nanotubes	Zn(II)	86.95						[221]
	Maghemite nanotubes	Cu(II)	111.11						[221]
	EDTA-modified chitosan/SiO_2_/Fe_3_O_4_	Cu(II)	0.495 mmol/g		720	5	25	12	[222]
	EDTA-modified chitosan/SiO_2_/Fe_3_O_4_	Pb(II)	0.045 mmol/g		720	5	25	12	[222]
	EDTA-modified chitosan/SiO_2_/Fe_3_O_4_	Cd(II)	0.040 mmol/g		720	5	25	12	[222]
	Fe_3_O_4_@mesoporous SiO_2_ core-shell	Pb(II)	128.21					6	[223]
	Fe_3_O_4_@mesoporous SiO_2_ core-shell	Cu(II)	51.81					6	[223]
	Fe_3_O_4_/GO	Cr(VI)	32.33			4.5	20		[224]
	Hollow nestlike α-Fe_2_O_3_ spheres	As(V)	75.3	88	120				[225]
	Hollow nestlike α-Fe_2_O_3_ spheres	Cr(VI)	58.6	67	120				[225]
	α-Fe_2_O_3_ nanofibers	Cr(VI)	16.17				25	4	[226]
	Fe_3_O_4_@SiO_2_–NH_2_	Pb(II)	243.9		180	5.2	25	5	[227]
	Cyanex-301-coated SPION	Cr(VI)	30.8			2	23		[228]
2014	S-doped Fe_3_O_4_@C	Cu(II)	54.7			5	35	4	[229]
	Fe_3_O_4_@SiO_2_-QTPA	Cd(II)		95.29	720		25		[230]
	Fe_3_O_4_@SiO_2_-QTPA	Zn(II)		92.37	720		25		[230]
	Fe_3_O_4_@SiO_2_-QTPA	Cu(II)		91.06	720		25		[230]
	Glycine-functionalized maghemite nanoparticles	Cu(II)	625			6.5	25		[231]
	Maghemite (γ-Fe_2_O_3_)	Pb(II)	10.55			7.5	45		[232]
	Maghemite (γ-Fe_2_O_3_)	Zn(II)	4.79			7.5	45		[232]
	Maghemite (γ-Fe_2_O_3_)	Cd(II)	1.75			7.5	45		[232]
	α-Fe_2_O_3_	Cr(VI)	17		300				[233]
	3-MPA-coated SPION	Cr(VI)	45			1	25		[234]
	EDA-Fe_3_O_4_ NPs	Cr(VI)		98	120	2		6	[235]
	M-FeHT	As(V)	1.2813		15	9	25		[236]
	M-FeHT	As(III)	0.1213		15	9	25		[236]
	Fe_3_O_4_-GS	Cr(VI)	17.29		240	1–3.5		5	[237]
	Fe_3_O_4_-GS	Pb(II)	27.95		120	6–7		5	[237]
	Fe_3_O_4_-GS	Hg(II)	23.03		120	6–7			[237]
	Fe_3_O_4_-GS	Cd(II)	27.83		120	6–7			[237]
	Fe_3_O_4_-GS	Ni(II)	22.07		120	6–7			[237]
	Fe_3_O_4_@CPS	Cu(II)	53.6			5	25	3	[238]
	Fe_3_O_4_@CPS	Cd(II)	87.1			6	25	3	[238]
	Fe_3_O_4_@CPS	Pb(II)	25.2			6	25	3	[238]
	MWCNT CoFe_2_O_4_–NH_2_	Pb(II)	140.1			6		5	[239]
	Fe_3_O_4_/talc	Cu(II)		72.15					[240]
	Fe_3_O_4_/talc	Ni(II)		50.23					[240]
2014	Fe_3_O_4_/talc	Pb(II)		91.35					[240]
	Magnetite nanoparticles	Cr(VI)	121.9		60	5.5			[241]
	CoFe_2_O_4_-rGO	Pb(II)	299.4		80	5.3	25		[242]
	CoFe_2_O_4_-rGO	Pb(II)	274.7				35		[242]
	CoFe_2_O_4_-rGO	Pb(II)	253.2				45		[242]
	CoFe_2_O_4_-rGO	Hg(II)	157.9		60	4.6	25		[242]
	CoFe_2_O_4_-rGO	Hg(II)	105.1				35		[242]
	CoFe_2_O_4_-rGO	Hg(II)	90.49				45		[242]
	Graphene Oxide−MnFe_2_O_4_	Pb(II)	673			5			[243]
	MnFe_2_O_4_	Pb(II)	488			5	60		[243]
	Graphene Oxide−MnFe_2_O_4_	As(III)	146			6.5	60		[243]
	MnFe_2_O_4_	As(III)	97			6.5	60		[243]
	Graphene Oxide−MnFe_2_O_4_	As(V)	207			4	60		[243]
	MnFe_2_O_4_	As(V)	136			4	60		[243]
	Water-soluble Fe_3_O_4_	Hg(II)		>99	10	7	25	3	[244]
	Fe_3_O_4_@C	Pb(II)	90.7	96.3	10				[245]
	Fe_3_O_4_@C	Hg(II)	83.1	98.1	10				[245]
	Fe_3_O_4_@C	Cd(II)	39.7	93.8	10				[245]
	Fe_3_O_4_@silica xanthan gum	Pb(II)	21.32			6	20	22	[246]
2015	Fe_3_O_4_-NTA	Cu(II)	40.24		35	5			[160]
	Magnetic chitosan/cellulose hybrid microspheres by embedding γ-Fe_2_O_3_	Cu(II)	88.21				30		[247]
	Magnetic chitosan/cellulose hybrid microspheres by embedding γ-Fe_2_O_3_	Cd(II)	61.1				30		[247]
	Magnetic chitosan/cellulose hybrid microspheres by embedding γ-Fe_2_O_3_	Pb(II)	45.86				30		[247]
	Mesoporous CoFe_2_O_4_	Pb(II)	32.11		480	5			[248]
	MAMNPs	Cd(II)	91.5		100	6		3	[249]
	MAMNPs	Hg(II)	237.6		100	6		3	[249]
	MAMNPs	Pb(II)	118.5		100	6		3	[249]
	Fe_3_O_4_/MMT NC	Pb(II)	263.15		2				[170]
	Fe_3_O_4_/MMT NC	Cu(II)	70.92		2				[170]
	Fe_3_O_4_/MMT NC	Ni(II)	65.78		2				[170]
	Fe_3_O_4_@SiO_2_ core-shell nanoparticles	Cd(II)	179						[250]
	Fe_3_O_4_@SiO_2_ core-shell nanoparticles	Pb(II)	156						[250]
	Fe_3_O_4_	Ni(II)	362.318			8		5	[251]
	Magnetic PAAAM/ PAMPS	Zn(II)	289.12				25		[252]
	Magnetic PAAAM/ PAMPS	Cd(II)	385.2				25		[252]
	Hollow magnetite nanospheres	Cr(VI)	6.64			4	25		[253]
	Hollow magnetite nanospheres	Cr(VI)	7.31			4	35		[253]
	Hollow magnetite nanospheres	Cr(VI)	8.90			4	45		[253]
	Hollow magnetite nanospheres	Pb(II)	13.40			5	25		[253]
2015	Hollow magnetite nanospheres	Pb(II)	14.11			5	35		[253]
	Hollow magnetite nanospheres	Pb(II)	18.47			5	45		[253]
	SPION	As(V)	0.91 mmol/g		60	3.8			[254]
	SPION surface-coated with 3-mercaptopropionic acid (3-MPA)	As(V)	1.92 mmol/g		60	3.6			[254]
	Fe_3_O_4_@SiO_2_	Cd(II)	24.8		180	7	25	5	[255]
	PPY/γ-Fe_2_O_3_	Cr(VI)	209		15	2		4	[256]
	PANI/γ-Fe_2_O_3_	Cr(VI)	196		35	2		4	[256]
	PPY/γ-Fe_2_O_3_	Cu(II)	171		15	5.5		4	[256]
	PANI/γ-Fe_2_O_3_	Cu(II)	107		35	5.5		4	[256]
	EDTA-Fe_3_O_4_	Pb(II)	508.4		40	4.2	45		[257]
	EDTA-Fe_3_O_4_	Hg(II)	268.4		50	4.1	45		[257]
	EDTA-Fe_3_O_4_	Cu(II)	301.2		90	5.1	45		[257]
	EDTA-Fe_3_O_4_	Pb(II)	548.1				35		[257]
	EDTA-Fe_3_O_4_	Hg(II)	242.2				35		[257]
	EDTA-Fe_3_O_4_	Cu(II)	289.4				35		[257]
	EDTA-Fe_3_O_4_	Pb(II)	481.2				25	5	[257]
	EDTA-Fe_3_O_4_	Hg(II)	203.1				25	5	[257]
	EDTA-Fe_3_O_4_	Cu(II)	246.1				25	5	[257]
	Fe_3_O_4_@SiO_2_/Schiff	Cu(II)	97.2		60	5			[258]
	Fe_3_O_4_@SiO_2_/Schiff	Zn(II)	81.6		60	5			[258]
	PMMA-gft-Alg/Fe_3_O_4_	Pb(II)	62.5			5	50		[259]
	PMMA-gft-Alg/Fe_3_O_4_	Cu(II)	35.71			5	50		[259]
	SH-HMSMCS	Hg(II)		95		6.2		5	[260]
	Ppy–Fe_3_O_4_/rGO	Cr(VI)	293.3			3	45		[261]
	Ppy–Fe_3_O_4_/rGO	Cr(VI)	226.8			3	30		[261]
	Ppy–Fe_3_O_4_/rGO	Cr(VI)	180.8			3	20		[261]
	Co_0_._6_Fe_2_._4_O_4_	Pb(II)	80.32		30		45.18		[262]
	Co_0_._6_Fe_2_._4_O_4_	Pb(II)	70.22				35.15		[262]
	Co_0_._6_Fe_2_._4_O_4_	Pb(II)	44.58				25.15		[262]
	Fe_3_O_4_/Mg–Al–CO_3_	Cd(II)	54.7				50		[263]
	Fe_3_O_4_/Mg–Al–CO_3_	Cd(II)	50.5				40		[263]
	Fe_3_O_4_/Mg–Al–CO_3_	Cd(II)	45.6				30		[263]
2016	NMag–CS	Cu(II)	123.4		120	5.5			[264]
	NMag–CS	Pb(II)	114.9		120	5.5			[264]
	NMag–CS	Cr(VI)	116.2		120	5.5			[264]
	NMag–CS	Cd(II)	112.3		120	5.5			[264]
	NMag–CS	Ni(II)	109.8		120	5.5			[264]
	Amino-functionalized Fe_3_O_4_@SiO_2_	Zn(II)	169.5		120	5 ± 0.1	25		[265]
	Fe_3_O_4_	Cu(II)	37.04			4–6			[266]
	Fe_3_O_4_	Pb(II)	166.67			4–6			[266]
	Fe_2_O_3_	Cu(II)	19.61			5–6		4	[266]
	Fe_2_O_3_	Pb(II)	47.62			3–4		4	[266]
	Fe_2_O_3_@SiO_2_	As(III)	77.7		180	7	30		[267]
2016	Fe_2_O_3_@TiO_2_	As(V)	99.5		180	6	30		[267]
	Fe_3_O_4_@SiO_2_-SH	Hg(II)	132			6	25	5	[268]
	Fe_3_O_4-_Aspa	Ni(II)	87.183		70	6	40		[269]
	Fe_3_O_4_-Aspa	Ni(II)	58.582		70	6	35		[269]
	Fe_3_O_4_-Aspa	Ni(II)	34.602		70	6	30	6	[269]
	Henna-Fe_3_O_4_	Cu(II)	28.90		20	5.2			[270]
	Fe_3_O_4_/Bent-2.0	Pb(II)	81.5		30				[271]
	Fe_3_O_4_/Bent-2.0	Cd(II)	21.7		30				[271]
	Fe_3_O_4_/Bent-2.0	Cu(II)	19.6		30				[271]
	Fe_3_O_4_	Pb(II)		100	30	5	25		[272]
	Fe_3_O_4_	Cr(VI)	34.9			2	45		[273]
	Fe_3_O_4_	Pb(II)	53.1			5	45		[273]
	Fe_3_O_4_	Cr(VI)	26.8			2	35		[273]
	Fe_3_O_4_	Pb(II)	52.8			5	35		[273]
	Fe_3_O_4_	Cr(VI)	20.2			2	25	2	[273]
	Fe_3_O_4_	Pb(II)	52.9			5	25	2	[273]
	a-Fe_2_O_3_	Cd(II)	127.23			6	20		[274]
	a-Fe_2_O_3_	Cd(II)	146.41			6	30		[274]
	a-Fe_2_O_3_	Cd(II)	158.48			6	40		[274]
	GO/Fe_3_O_4_	Pb(II)	65.96		180	3	20		[275]
	GO/Fe_3_O_4_/LA	Pb(II)	53.06		180	3	20		[275]
	GO/Fe_3_O_4_/LA/EDTA	Pb(II)	161.80		180	3	20		[275]
	Amine-functionalized Fe_3_O_4_	Cu(II)		85	30	7			[276]
	Fe_3_O_4_@SiO_2_@TiO_2_	Cu(II)	125		30				[277]
	Fe_3_O_4_@SiO_2_@TiO_2_	Zn(II)	137		30				[277]
	Fe_3_O_4_@SiO_2_@TiO_2_	Cd(II)	148		30				[277]
	Fe_3_O_4_@SiO_2_@TiO_2_	Pb(II)	160		30				[277]
	Rice straw/Fe_3_O_4_ NCs	Pb(II)		91.18					[278]
	Rice straw/Fe_3_O_4_ NCs	Cu(II)		75.54					[278]
	Cys-Fe_3_O_4_	Pb(II)	183.5		120	6		5	[279]
	Cys-Fe_3_O_4_	Cd(II)	64.35		120	6		5	[279]
	Nano-Fe_3_O_4_@Nano-SiO_2_	Pb(II)	1100 μmol/g						[280]
	Nano-Fe_3_O_4_@Nano-SiO_2_	Cu(II)	300 μmol/g						[280]
	Nano-Fe_3_O_4_@Nano-SiO_2_	Cd(II)	150 μmol/g						[280]
	Nano-Fe_3_O_4_@Nano-SiO_2_	Hg(II)	100 μmol/g						[280]
	a-Fe_2_O_3_	As(V)	38.48				20		[281]
	Fe_3_O_4_-MnO_2_	Pb(II)	208.17				25		[282]
	Fe_3_O_4_-MnO_2_	Cu(II)	111.90				25		[282]
	Fe_3_O_4_-MnO_2_	Cd(II)	169.90				25		[282]
	Fe_3_O_4_-MnO_2_	Zn(II)	100.24				25		[282]
	Fe_3_O_4_-MnO_2_	Ni(II)	55.63				25		[282]
	Fe_3_O_4_@SiO_2_-EDTA	Cu(II)	37.59		10	5.3	30		[283]
	Fe_3_O_4_@SiO_2_-EDTA	Pb(II)	114.94		10	5.3	30		[283]
	Fe_3_O_4_@SiO_2_-EDTA	Ni(II)	32.15		10	5.3	30		[283]
	Fe_3_O_4_@SiO_2_-EDTA	Cd(II)	50.25		10	5.3	30		[283]
	Fe_3_O_4_–FeB	Cr(VI)	38.9			6.3			[284]
	Fe_3_O_4_–TiO_2_	As(V)	11.434 μg/g		40	6.3	25		[285]
	Fe_3_O_4_–TiO_2_	As(III)		93		11	25		[285]
	Chitosan MWCNT/Fe_3_O_4_	Cr(VI)	360.1		30	2	45		[286]
2016	Chitosan MWCNT/Fe_3_O_4_	Cr(VI)	348.2				35		[286]
	Chitosan MWCNT/Fe_3_O_4_	Cr(VI)	335.6				25		[286]
	AMGO	Cr(VI)	123.4			2		5	[287]
2017	Fe_3_O_4_@MnO_2_	Pb(II)	666.67		120		25		[148]
	Fe_3_O_4_-SO_3_H	Pb(II)	108.93			7	25		[288]
	Fe_3_O_4_-SO_3_H	Cd(II)	80.9			7	25		[288]
	PGMA-MAn Copolymer@Fe_3_O_4_	Pb(II)	53.33		20	5	25		[289]
	PGMA-MAn Copolymer@Fe_3_O_4_	Cu(II)	48.53			5			[289]
	Fe_3_O_4_/CTAB	Cr(VI)		95.77	720	4	25 ± 0.1		[290]
	Fe_3_O_4_@Cu_3_(btc)_2_	Pb(II)	215.05					4	[291]
	Fe_3_O_4_@Cu_3_(btc)_2_	Hg(II)	348.43					4	[291]
	Fe_3_O_4_-CS-L	Zn(II)	256.41		45	6	25	5	[292]
	Fe_3_O_4_-CS-L	Cd(II)	156.99		45	6	25	5	[292]
	Fe_3_O_4_-CS-L	Pb(II)	128.63		45	6	25	5	[292]
	Fe_3_O_4_-mSiO_2_	Cu(II)	84.4						[293]
	Fe_3_O_4_-mSiO_2_	Cd(II)	80.5						[293]
	Fe_3_O_4_-mSiO_2_	Zn (II)	72.6						[293]
	γ-Fe_2_O_3_@CS	Cd(II)	15.2		60	5	20 ± 0.1	5	[294]
	L-Cyst-Fe_3_O_4_	Pb(II)	18.8			2	45	5	[295]
	L-Cyst-Fe_3_O_4_	Cr(VI)	34.5			6	45	5	[295]
	MDA-Fe_3_O_4_	Pb(II)	333.3			5	30		[296]
	ZrO_2_-Fe_3_O_4_	As(III)	113.48			7		5	[297]
	Fe_3_O_4_@SiO_2_@CS-TETA-GO	Cu(II)	324.7		16	6	30	6	[298]
	CoFe_2_O_4_@SiO_2_–SH	Hg(II)	641.0			8	25	5	[299]
	CoFe_2_O_4_@SiO_2_–SH	Hg(II)	628.9			8	35		[299]
	CoFe_2_O_4_@SiO_2_–SH	Hg(II)	591.7			8	45		[299]
	EDTA-functionalized CoFe_2_O_4_ (EDTA-MNP)	Cu(II)	73.26			6	50		[300]
	IMSA	Pb(II)	133.73						[301]
	IMSA	As(V)	21.61						[301]
	CoFe_2_O_4_@SiO_2_	Cd(II)	199.9			7		5	[302]
	CoFe_2_O_4_@SiO_2_	Cu(II)	177.8			7		5	[302]
	CoFe_2_O_4_@SiO_2_	Pb(II)	181.6		30	7		5	[302]
	TEPA chitosan/CoFe_2_O_4_	Cu(II)	168.067		50	5	30		[105]
	TEPA chitosan/CoFe_2_O_4_	Pb(II)	228.311		50	5	30		[105]
2018	Lantana camara capped iron nanoparticles	Ni(II)	227.2			6	60		[303]
	NA-FeO_x_	Cd(II)	11.3		60				[304]
	NA-FeO_x_	Cu(II)	12.3		60				[304]
	Graphene oxide-Fe_3_O_4_	Pb(II)	373.14		10	6			[305]
	PAE-AAm-g-MNPs	Cu(II)	30.34			6		7	[306]
	Fe_3_O_4_/LDH-AM	Cu(II)	64.66		240			5	[307]
	Fe_3_O_4_/LDH-AM	Cd(II)	74.06		240			5	[307]
	Fe_3_O_4_/LDH-AM	Pb(II)	266.6		180			5	[307]
	TEPA-GO/MnFe_2_O_4_	Pb(II)	263.2			5.5	30	4	[308]
	GO/MnFe_2_O_4_	Pb(II)	133.3						[308]
	MnFe_2_O_4_–BC	Cd(II)	181.49			7	25	5	[309]
2018	CoFe_2_O_4_@SiO_2_	Hg(II)	149.3			7	25	5	[310]
	CoFe_2_O_4_@SiO_2_	Hg(II)	144.9			7	35		[310]
	CoFe_2_O_4_@SiO_2_	Hg(II)	131.6			7	45		[310]
	p-BNMR@Fe_3_O_4_	Pb(II)	249.5			5.5	25		[311]
	Aminated-Fe_3_O_4_	Cr(VI)	19.5			3.5		3	[312]
	Aminated-Fe_3_O_4_	Pb(II)	21.2			3.5		3	[312]
	Fe_3_O_4_/poly(C_3_N_3_S_3_)	Pb(II)	232.6			6	25	7	[313]
	Fe_3_O_4_/poly(C_3_N_3_S_3_)	Hg(II)	344.8			6	25	7	[313]
	rGO-PDTC/Fe_3_O_4_	Cu(II)	113.64			5	25	5	[314]
	rGO-PDTC/Fe_3_O_4_	Cd(II)	116.28			6	25	5	[314]
	rGO-PDTC/Fe_3_O_4_	Pb(II)	147.06			6	25	5	[314]
	rGO-PDTC/Fe_3_O_4_	Hg(II)	181.82			6	25	5	[314]
	d-MoS2/Fe_3_O_4_	Hg(II)	425.5		20			5	[315]
	CHT/ALG/Fe_3_O_4_@SiO_2_ (8 beads)	Pb(II)	243.77			4.2	20		[316]
	CHT/ALG/Fe_3_O_4_@SiO_2_ (1 bead)	Pb(II)	228.73			4.2	20		[316]
2019	Fe_3_O_4_/BC/AC	Pb(II)	169.78			5	25	5	[317]
	Fe_3_O_4_@FePO_4_	Cd(II)	13.51		15	7			[318]
	Fe_3_O_4_@SiO_2_-NH-MFL	Pb(II)	150.33		0.5	5			[156]
	Fe_3_O_4_@SiO_2_-NH-MFL	Cu(II)	70.7		0.5	5			[156]
	DTT-Fe_3_O_4_@Au	As(III)		68.8		5			[319]
	rGO-poly(C_3_N_3_S_3_)/Fe_3_O_4_	Pb(II)	270.3		60	6	25	15	[320]
	rGO-poly(C_3_N_3_S_3_)/Fe_3_O_4_	Hg(II)	400		60	6	25	15	[320]
	APTES-Fe_3_O_4_ (3 wt%)	As(V)	14.6		210	2	25		[321]
	Fe_2_O_3_-SiO_2_-PAN	Cr(III)	4.36						[322]
	Fe_2_O_3_-SiO_2_-PAN	Cu(II)	7.20						[322]
	Fe_2_O_3_-SiO_2_-PAN	Zn(II)	5.06						[322]
	Fe_2_O_3_-SiO_2_-PAN	Ni(II)	2.60						[322]
	biochar-MnFe_2_O_4_	Pb(II)	154.94			5	25		[323]
	biochar-MnFe_2_O_4_	Cd(II)	127.83			5	25		[323]
	CoFe_2_O_4_@SiO_2_-EDTA	Hg(II)	103.3		360	7	25	5	[324]
	Fe_3_O_4_-CS@BT	Cr(VI)	62.1			2	25	5	[325]
	Fe_3_O_4_-CS@BT	Cr(VI)	48.3			4	25		[325]
	Fe_3_O_4_-CS@BT	Cr(VI)	36.4			6	25		[325]
	CMC/SA/graphene oxide@Fe_3_O_4_	Cu(II)	55.96			5	30		[326]
	CMC/SA/graphene oxide@Fe_3_O_4_	Cd(II)	86.28			6	30		[326]
	CMC/SA/graphene oxide@Fe_3_O_4_	Pb(II)	189.04			5	30		[326]
	Fe_3_O_4_/NaP/NH_2_	Pb(II)	181.81		480	5–6	60	10	[327]
	Fe_3_O_4_/NaP/NH_2_	Cd(II)	50.25		240	5–6	70	10	[327]
	Fe_3_O_4_ ECSBNC	Cu(II)	90.90						[328]
	Fe_3_O_4_ ECSBNC	Cr(VI)	83.33						[328]
	MNPs-COOH	Pb(II)	0.855 mmol/g			6	25		[329]
	MNPs-COOH	Cu(II)	0.660 mmol/g			6	25		[329]
2019	MNPs-COOH	Cd(II)	0.518 mmol/g			6	25		[329]
	MNPs-COOH	Ni(II)	0.441 mmol/g			6	25		[329]
	CMC-Fe_3_O_4_	Pb(II)	152.0					6	[330]
	Fe_3_O_4_-loaded CS NPs	Cd(II)	97.76			5		5	[331]
	CuFe_2_O_4_	Cd(II)	157.7			2			[332]
	CoFe_2_O_4_	Pb(II)	63.1			12			[332]
	HP-β-CD-GO/Fe_3_O_4_	Cu(II)	17.91			6		5	[333]
	HP-β-CD-GO/Fe_3_O_4_	Pb(II)	50.39			5		5	[333]
	Bentonite/CoFe_2_O_4_@MnO_2_-NH_2_	Cd(II)	115.79	98.88					[334]
2020	Fe_3_O_4_@SiO_2_@GLYMO(S)	Cd(II)	80.64		55	7		5	[335]
	Fe_3_O_4_@SiO_2_@GLYMO(S)	Pb(II)	93.5		55	7		5	[335]
	MGO	Pb(II)	200		30	5			[167]
	MGO	Cr(III)	24.33		30	6			[167]
	MGO	Cu(II)	62.89		30	6			[167]
	MGO	Zn(II)	63.69		30	7			[167]
	MGO	Ni(II)	51.02		30	8			[167]
	Proanthocyanidin- functionalized Fe_3_O_4_	Cu(II)	18.8		30	8		5	[336]
	Proanthocyanidin- functionalized Fe_3_O_4_	Cd(II)	20.9		30	8			[336]
	Proanthocyanidin-functionalized Fe_3_O_4_	Pb(II)	21.5		30	8			[336]
	Ggh-g-PAcM/Fe_3_O_4_	Cu(II)	224.8				30		[337]
	Ggh-g-PAcM/Fe_3_O_4_	Hg(II)	213.8				30		[337]
	Fe_3_O_4_-GO hybrid (9:1)	Pb(II)	107.56			6			[338]
	Fe_3_O_4_-GO hybrid (5:1)	Pb(II)	151.22			6			[338]
	M-45 OA	Pb(II)	42.553			6			[338]
	M-45 OA	Zn(II)	42.919			6			[339]
	M-45 OA	Cd(II)	42.373			7			[339]
	M-55 OA	Pb(II)	41.841			6			[339]
	M-55 OA	Zn(II)	42.735			6			[339]
	M-55 OA	Cd(II)	42.017			7			[339]
	M-55+	Pb(II)	40.816			6			[339]
	M-55+	Zn(II)	40.816			6			[339]
	M-55+	Cd(II)	39.216			7			[339]
	BNNF@Fe_3_O_4_	Pb(II)	203.75		50		25		[340]
	Fe_3_O_4_@SiO_2_-NH_2_	Cd(II)		93				5	[341]
	RH + iron oxide NPs	As(V)	82		60			5	[342]
	MnFe_2_O_4_	Zn(II)	454.4		120	6	25	3	[343]
	CoFe_2_O_4_	Zn(II)	384.6		120	6	25	3	[343]
	SiO_2_/CuFe_2_O_4_/PANI	Cu(II)	285.71		300	5.3	30	4	[344]
	MWCNT/γ-Fe_2_O_3_	Cr(VI)	208.1		150	4			[345]
	MWCNT-PEI/γ-Fe_2_O_3_	Cr(VI)	352.3		150	4			[345]
	Fe_3_O_4_	Cr(VI)	201.55		50			6	[346]
	Fe_3_O_4_@Z-NCNT/PC	Pb(II)	789.87		20	5.5		10	[347]
	FO-BC-450	Cd(II)	151.3			2	25		[348]
2020	FO-BC-450	Cu(II)	219.8			2	25		[348]
	FO-BC-450	Pb(II)	271.9			2	25		[348]
	Fe_2_O_3_@SiO_2_@SH	Hg(II)		98					[349]
	γ-Fe_2_O_3_ coated Bacillus subtilis	Cd(II)	32.6			4	30		[350]
	Fe_3_O_4_-HBPA-ASA	Cu(II)	136.66			8	25	5	[351]
	Fe_3_O_4_-HBPA-ASA	Cd(II)	88.36			8	25	5	[351]
	Fe_3_O_4_-HBPA-ASA	Pb(II)	165.46			8	25	5	[351]
	Crystalline iron oxide nanoparticles (IO-NPs)	Pb(II)	9.206			6	40		[352]
	Crystalline iron oxide nanoparticles (IO-NPs)	Ni(II)	9.666			6	40		[352]
	Crystalline iron oxide nanoparticles (IO-NPs)	Cu(II)	8.355			6	40		[352]
	Crystalline iron oxide nanoparticles (IO-NPs)	Zn(II)	9.106			6	40		[352]
	Pec-g-PHEAA/Fe_3_O_4_	Cu(II)	248.6						[353]
	Pec-g-PHEAA/Fe_3_O_4_	Hg(II)	240.2						[353]
	Fe_3_O_4_ fibre	Pb(II)	16.78					6	[354]
	Fe_3_O_4_ powder	Pb(II)	15.80					6	[354]
	Iron oxide (MNPs) grafted (HPG)	Cu(II)	0.700 mg/mg		120	9	20		[355]
	Iron oxide (MNPs) grafted (HPG)	Ni(II)	0.451 mg/mg		120	9	20		[355]
	Nano-CI	Pb(II)	1900 μmol/g		30	6			[356]
	Nano-CI	Cu(II)	2250 μmol/g		30	7			[356]
	Nano-CI	Cd(II)	850 μmol/g		30	7			[356]
	Nano-CIC	Pb(II)	2700 μmol/g		30	4			[356]
	Nano-CIC	Cu(II)	4250 μmol/g		30	6			[356]
	Nano-CIC	Cd(II)	1800 μmol/g		30	6			[356]
	Nano-CIS	Pb(II)	2600 μmol/g		10	4			[356]
	Nano-CIS	Cu(II)	4700 μmol/g		10	6			[356]
	Nano-CIS	Cd(II)	1900 μmol/g		10	6			[356]

^1^ Cases with unit of adsorption capacity that is different from mg/g are listed next to the respective value.

**Table 6:** Recycling efficiency (%).

Pollutant	1st Cycle	2nd Cycle	3rd Cycle	4th Cycle	5th Cycle	Ref.
Pb(II)	90.12	88.05	85.65	81.35		[360]
Cu(II)	93.70				58.66	[196]
Pb(II)					90	[197]
Cr(VI)	55	88		90		[361]
Pb(II)	97.34				90	[175]
Hg(II)					≥96	[157]
Pb(II)					90	[201]
Pb(II)	93.5				89.3	[335]
Cu(II)	80.64				73.3	[335]
As(V)	95				56	[342]
Cd(II)				76.4		[348]
Cu(II)				80.4		[348]
Pb(II)				70.2		[348]
Hg(II)	>90				~75.5	[324]
Cd(II)	98.8	95.1	91.7	84.6	78.3	[294]
Cd(II)	99.96			97.25		[302]
Cu(II)	88.05			84.15		[302]
Pb(II)	90.79			87.12		[302]
Pb(II)	96.2				86.4	[257]
Hg(II)	95.1				85.9	[257]
Cu(II)	96.5				87.6	[257]

**Table 7:** Recycling adsorption capacity (mg/g).

Pollutant	1st Cycle	2nd Cycle	3rd Cycle	4th Cycle	5th Cycle	6th Cycle	Ref.
Cr(VI)	132.56	130.62	127.52	125.97	124.42	121.71	[346]
Cu(II)	197.5	196.5	195	194.4			[344]

## Data Availability

Data is contained within the article and references therein.
